# Comparative virome analysis of individual shedding routes of *Miniopterus phillipsi* bats inhabiting the Wavul Galge cave, Sri Lanka

**DOI:** 10.1038/s41598-023-39534-3

**Published:** 2023-08-08

**Authors:** Therese Muzeniek, Thejanee Perera, Sahan Siriwardana, Dilara Bas, Fatimanur Bayram, Mizgin Öruc, Beate Becker-Ziaja, Inoka Perera, Jagathpriya Weerasena, Shiroma Handunnetti, Franziska Schwarz, Gayani Premawansa, Sunil Premawansa, Wipula Yapa, Andreas Nitsche, Claudia Kohl

**Affiliations:** 1 Centre for Biological Threats and Special Pathogens, Highly Pathogenic Viruses (ZBS 1), Robert Koch Institute, 13353 Berlin, Germany; 2 Institute of Biochemistry, Molecular Biology and Biotechnology, University of Colombo, Colombo, 00300 Sri Lanka; 3 IDEA (Identification of Emerging Agents) Laboratory, Department of Zoology and Environment Sciences, University of Colombo, Colombo, 00300 Sri Lanka; 4 Centre for International Health Protection, Public Health Laboratory Support (ZIG 4), Robert Koch Institute, 13353 Berlin, Germany; 5 Colombo North Teaching Hospital, Ragama, 11010 Sri Lanka

**Keywords:** Biodiversity, Molecular biology

## Abstract

Bats are described as the natural reservoir host for a wide range of viruses. Although an increasing number of bat-associated, potentially human pathogenic viruses were discovered in the past, the full picture of the bat viromes is not explored yet. In this study, the virome composition of *Miniopterus phillipsi* bats (formerly known as* Miniopterus fuliginosus *bats in Sri Lanka) inhabiting the Wavul Galge cave, Sri Lanka, was analyzed. To assess different possible excretion routes, oral swabs, feces and urine were collected and analyzed individually by using metagenomic NGS. The data obtained was further evaluated by using phylogenetic reconstructions, whereby a special focus was set on RNA viruses that are typically associated with bats. Two different alphacoronavirus strains were detected in feces and urine samples. Furthermore, a paramyxovirus was detected in urine samples. Sequences related to *Picornaviridae*, *Iflaviridae*, unclassified *Riboviria* and *Astroviridae* were identified in feces samples and further sequences related to *Astroviridae* in urine samples. No viruses were detected in oral swab samples. The comparative virome analysis in this study revealed a diversity in the virome composition between the collected sample types which also represent different potential shedding routes for the detected viruses. At the same time, several novel viruses represent first reports of these pathogens from bats in Sri Lanka. The detection of two different coronaviruses in the samples indicates the potential general persistence of this virus species in *M.* *phillipsi* bats. Based on phylogenetics, the identified viruses are closely related to bat-associated viruses with comparably low estimation of human pathogenic potential. In further studies, the seasonal variation of the virome will be analyzed to identify possible shedding patterns for particular viruses.

## Introduction

Bats are species-rich and taxonomically diverse mammals in the order *Chiroptera* that are distributed worldwide^1^. They represent a large group (20%) of mammals and share unique features like their ability to fly. A number of viruses from different viral families, including human pathogenic viruses like Hendra and Nipah virus, coronaviruses, lyssaviruses and others, have been associated with bats^2^. It is assumed that these viruses evolved together with their natural reservoir hosts. Because of this co-speciation and adaptation process, the viruses are often less pathogenic for their bat hosts. It is assumed that the bats’ immune system is adapted to virus infections and hence able to control them without developing visible symptomatic diseases^3–5^.

With the increasing focus on bat virus research, the detection of potentially zoonotic viruses with species-specific or family-specific PCR assays (e.g., paramyxoviruses, lyssaviruses and coronaviruses) has been a convenient standard method. However, conventional detection methods are reliant on primers designed from known diversity, and this may lead to bias focusing only on certain viruses or on those of particular interest^6–9^. To investigate the unknown viral diversity that may be present within the host species, metagenomic NGS methods (mNGS) for virus discovery can allow for untargeted and more unbiased sequencing of novel viruses. The analysis of the viral composition from bat samples (viromes) allows to reduce the bias and to constantly increase the number of viral sequences deposited in sequence databases such as GenBank of the National Center for Biotechnology Information (NCBI)^10–12^.

However, in several regions of the world the investigation of bats in their role as potential reservoir host of zoonotic viruses has been barely conducted^6^. In Sri Lanka, zoology is an important research field and ecological aspects of bats are well investigated^13^. Bats significantly contribute to the biodiversity and account for about a third of the Sri Lankan mammals with 30 different species^14^. Furthermore, they are essential for the maintenance of the ecosystem by providing ecoservices such as pollination, seed dispersal and insect control^13^.

In contrast, only few studies have focused on bats as reservoir host for pathogens in Sri Lanka^15–18^. Here we present the first virome analysis of *M.* *phillipsi* bats inhabiting the Wavul Galge cave (Koslanda, Sri Lanka) in the interior of Sri Lanka. *M.* *phillipsi* bats are roosting sympatrically with the other bat species *Hipposideros lankadiva, Hipposideros speoris, Rhinolophus rouxii* and *Rousettus leschenaultii*. In three individual field studies at different time points, we captured bats of all representative species and collected different sample material depending on availability^19^. Selected sample sets had been analyzed in previous investigations focusing on different research questions^17,18,20,21^. In this study, we focus on the virome analysis of urine swabs (US), oral swabs (OS) and feces (F) collected from *M.* *phillipsi* bats at one sampling point (July 2018). The presented results give first insights into the virome composition of this bat species in the Wavul Galge cave and in general. Furthermore, the results may point to differences in viral shedding routes by analyzing the different sample types. This study is the stepping stone for the further investigation of selected viruses in this species and cave.

## Methods

The study was approved by the local government authority (Department of Wildlife Conservation, Sri Lanka, permit No. WL/3/2/05/18, issued on 10 January 2018). Catching and sampling of bats was carried out according to previously assessed, established and described standard procedures to maximize the safety of the research group and to minimize the stress for the bats during the sampling procedure^19^. We adhered to relevant guidelines and regulations of the Fauna and Flora Protection Ordinance.

### Bat sampling

Sampling of bats inhabiting the Wavul Galge cave (Sri Lanka) was performed in March and July 2018 and January 2019 as described before^19^. For the results presented in this study, a subset of samples was selected from 188 *M. phillipsi* bats sampled in July 2018. Details about the sex, age status and morphometric measures taken from each bat are given in the supplementary material (Table ST1). Different sample types were collected depending on availability. From the selected subset of *M. phillipsi* bats a total of 187 oral swabs (OS) and 102 urine swabs (US) were collected with Minitip FLOQSwabs® (Copan Diagnostics, Murrieta, CA, USA); furthermore 77 fecal pellets (F) were collected with forceps. An overview of the collected samples per individual bat is given in the supplementary material (Table ST1).

**Figure 1:**
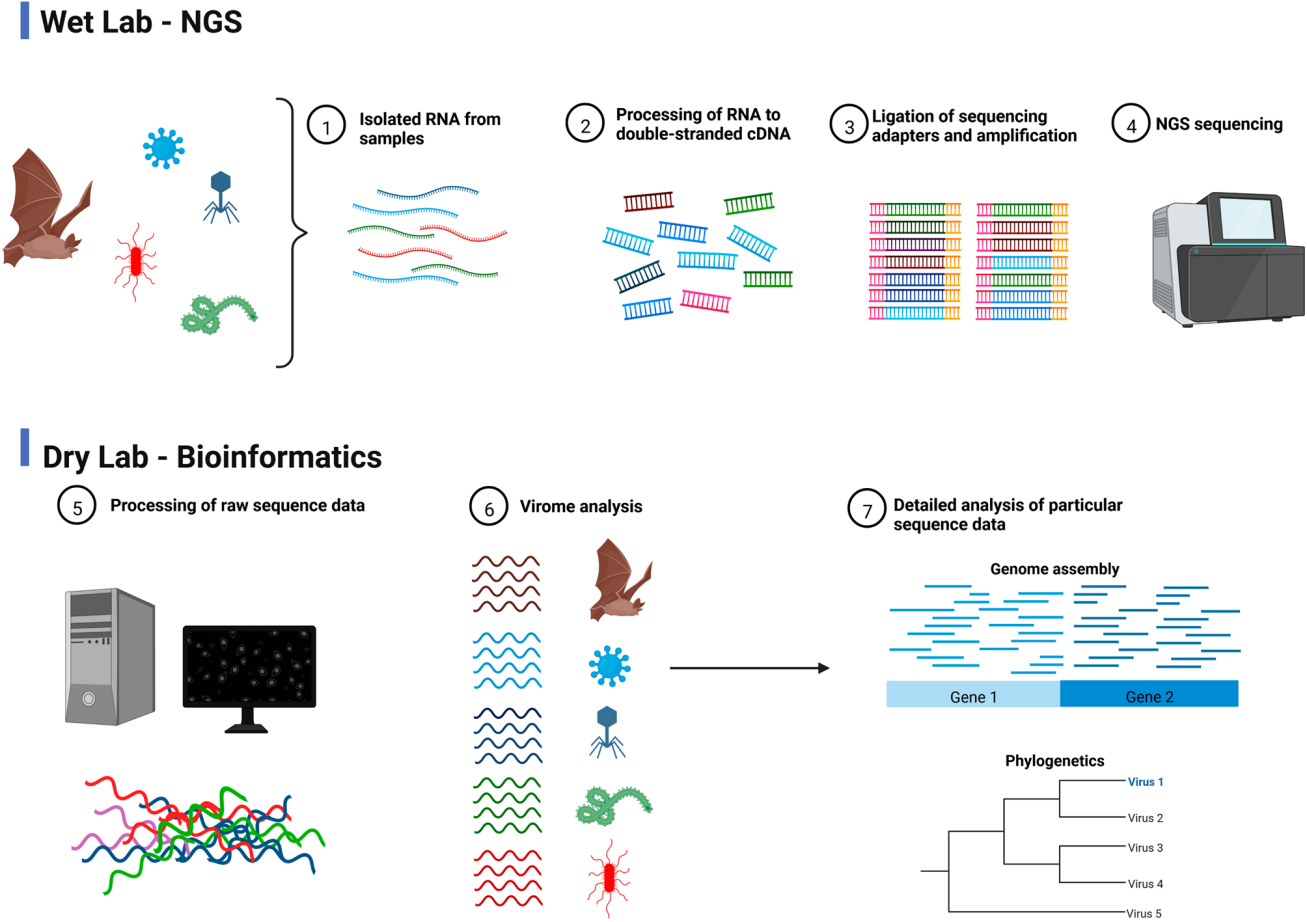
Schematic illustration of the general NGS workflow (wet lab) with subsequent bioinformatic analysis of obtained data (dry lab). Created with BioRender.com.

The samples were collected in cryotubes without any additives and then snap-frozen by using liquid nitrogen. After transportation samples were stored at − 80 °C until further processing. The general workflow of subsequent laboratory work and bioinformatic analysis of NGS data is shown in Fig. 1.

### Metagenomic NGS

The processing of samples was conducted with appropriate precautions in biosafety-2 laboratories. All samples were initially processed by adding 500 µL of sterile PBS. For OS and US, samples were mixed by vortexing before 140 µL were used for extraction with the Viral RNA Mini Kit according to the manufacturer’s instructions (QIAGEN, Hilden, Germany). Fecal pellets were homogenized with sterile ceramic beads by using the FastPrep-24 device for 60 s (3 cycles for 20 s at full speed and cooled on ice between cycles) (MP Biomedicals, Eschwege, Germany), followed by centrifugation for 5 min at 8000 × g and extraction of 140 µL of the supernatant with the Viral RNA Mini Kit according to the manufacturer’s instructions.

Prior to further processing a maximum of 10 RNA extracts were pooled by taking 5 µL per sample to obtain a final volume of 50 µL. Pools were prepared per sample type, resulting in 19 OS pools, 13 US pools and 8 F pools. The pooled RNA samples were treated with 4 U TURBO DNase for 30 min at 37 °C by using a TURBO DNA-free Kit (Invitrogen, Carlsbad, CA, USA), followed by inactivation of the TURBO DNase according to the manufacturer’s protocol. 20 µl of the treated RNA was transcribed into cDNA by using the SuperScript IV Reverse Transcriptase reagent (Invitrogen) and 50 µM random hexamer primers; the reaction was incubated at 23 °C for 10 min, 50 °C for 10 min and inactivated at 80 °C for 10 min. Second strand was synthesized for 1 h at 16 °C by using the NEBNext® Ultra™ II Non-Directional RNA Second Strand Synthesis Module (New England Biolabs, Ipswich, MA, USA) according to the manufacturer’s protocol. dsDNA was purified by using the Agencourt AMPure XP bead system (Beckman Coulter Life Sciences, Krefeld, Germany) following the manufacturer’s instructions, and dsDNA was eluted in 40 µL of PCR-grade H_2_O. The final DNA concentration was determined by using a NanoDrop™ 1000 Spectrophotometer (Thermo Fisher Scientific, Hennigsdorf, Germany). Sample libraries were sequenced on a HiSeq 2500 or a NextSeq 550 sequencing device (Illumina, San Diego, CA, USA) with a paired end read output of 2 × 250 bp (HiSeq) or 2 × 150 bp (NextSeq) and a total output of up to 8 million reads per pool. A detailed overview of the pools, included samples and obtained read output is given in the supplementary material (Table ST2).

### NGS data analysis and virome assembly

Prior to NGS data analysis, sequencing reads were trimmed and filtered by length and quality by using the tool Trimmomatic v0.39^22^. Surviving reads were mapped to the non-redundant protein virus database (NCBI, RefSeq release 210 from 3 January 2022) by using the diamond BLASTx algorithm (–sensitive) to^12,23^. The results and the distribution of assigned viral reads per pool were visualized in MEGAN^24^. This was also compared between the different sample types and among the pools of each sample type.

After obtaining a general overview in MEGAN, a selection of viral hits was further analyzed in detail. This selection focused mainly on RNA viruses that are known to be found in bats, humans or other mammals, but further viral hits with a high number of assigned reads were also selected for further investigation. Other viral hits that are typical sequencing contaminants or assigned to bacteriophages were not considered in the subsequent analysis.

The selected viral reads were extracted and assembled using Velvet with default quality settings to produce larger contigs^25^. These contigs were blasted to the NCBI database by using the BLASTn algorithm and default settings to identify the closest related sequence in the database. If available, the full genome sequence (with the highest identity on nt level) was downloaded to serve as reference. The initially trimmed NGS data was mapped to this reference to identify more reads and to evaluate the mapping quality visually in Geneious Prime software (version 2020.2.3, Biomatters Ltd., Auckland, New Zealand). Wherever possible, the nucleotide identities to related strains were calculated for the longest contig assembled on a conserved gene such as the RNA polymerase gene.

For all analyzed viral sequences of high interest, suitable primers were designed on contigs supported by high read coverage. Subsequently, the presence of the obtained viral sequences was confirmed in the initial individual cDNA samples by using conventional PCR under standard conditions (available on request). PCR products appearing as bands in the analytic agarose gel were purified and Sanger sequenced when sufficient quantity was reached.

### Phylogenetic reconstruction

For viral sequences of interest for this study, suitable contigs were used for phylogenetic reconstruction. To this end, preferably the longest contig on a conserved gene of the virus genome (e.g., RNA polymerase gene) was selected. A number of reference sequences were downloaded from NCBI database and an end-gap free nucleotide alignment was calculated by using the MAFFT algorithm^26^. The phylogenetic trees were calculated based on this alignment by using the Bayesian MCMC algorithm (MrBayes version 3.2.6)^27^. A suitable model for each alignment was estimated by using the Akaike information criterion prediction with the tool JModelTest^28,29^. The calculation parameters were different depending on the virus and are each specified in the results section (see legends for Figs. 4, 5 and 6).

### Ethical statement

The study was approved by the local government authority (Department of Wildlife Conservation, Sri Lanka, permit No. WL/3/2/05/18, issued on 10 January 2018). Catching and sampling of bats was carried out according to relevant guidelines and regulations of the Fauna and Flora Protection Ordinance, Sri Lanka. The results reported in this study were obtained without conducting animal experiments. All bat samples were collected non-invasively and bats were released after the sampling procedure. Relevant guidelines for animal research in vivo (ARRIVE) are not applicable.

## Results

**Figure 2:**
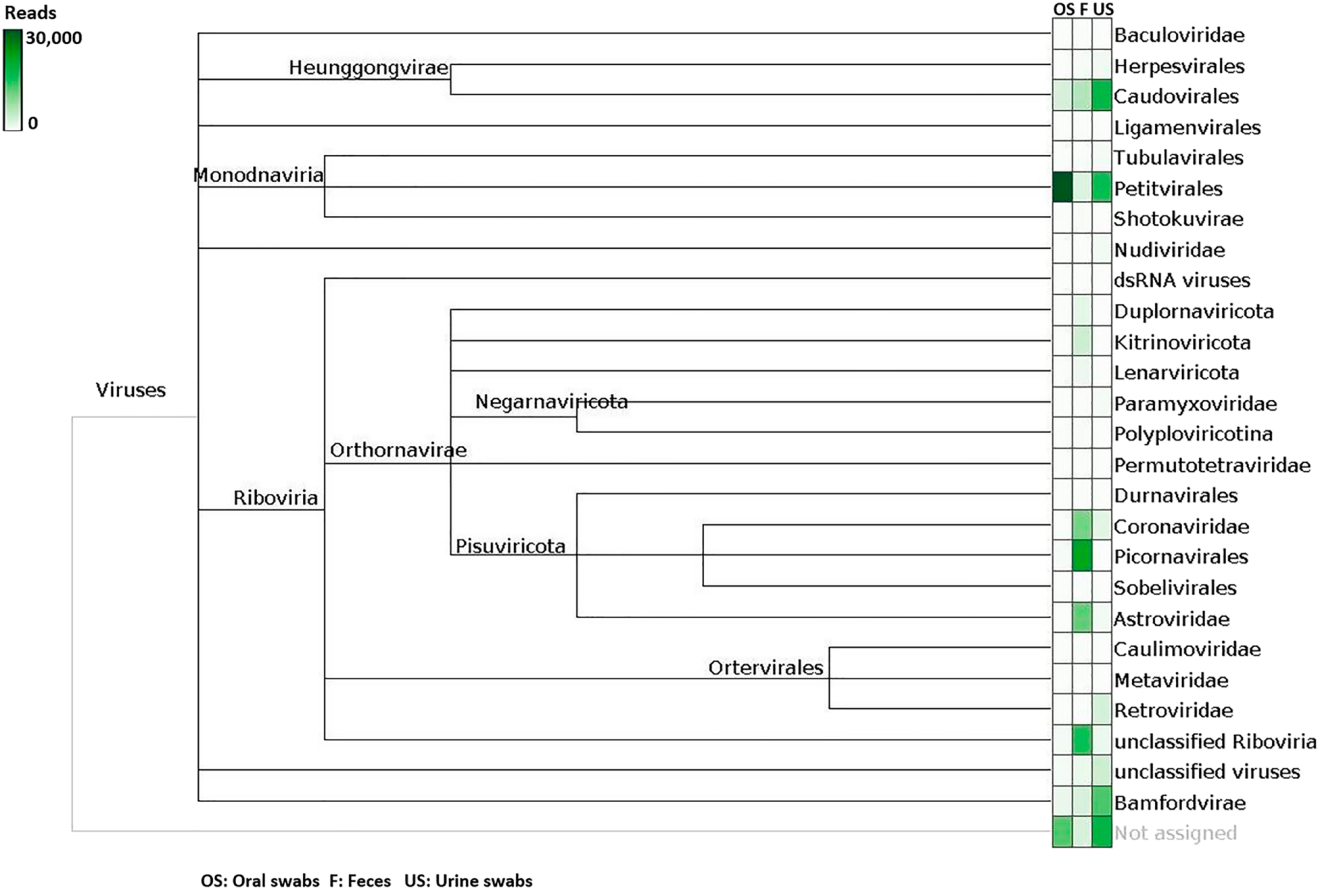
Normalized comparison of viral hits from different sample types in MEGAN after diamond BLASTx. Sample types are depicted in the following order: oral swabs (OS), feces (F) and urine swabs (US). The intensity of green color represents the quantity of reads assigned to the respective viral family or order.

Following mNGS, the sequence reads were trimmed and analyzed separately per pool as described in the methods section. Comparison of the pools per sample type revealed mainly homogenous distributions of viral reads assigned to the respective viral orders (see supplemental Figures SF1–SF3). For further analysis, viral reads of all pools per sample type were combined and the assigned viral reads compared between OS, F and US by using MEGAN as shown in Fig. 2.

As shown in Fig. 2, bacteriophages (e.g., *Caudovirales* and *Petitvirales*) were found in all sample types but in different amounts. For OS, no other viral hits of interest were assigned from the NGS data. Viral reads belonging to five different families (*Coronaviridae*, *Iflaviridae*, *Picornaviridae, Astroviridae* and *Paramyxoviridae*) and unclassified *Riboviria* were found in F samples and confirmed by mapping to the respective reference sequence and by PCR in the initial cDNA samples. In the US samples, viral reads of three families (*Paramyxoviridae*, *Coronaviridae* and *Astroviridae*) were detected and also confirmed by PCR in the initial cDNA samples.

Some of the other preliminary results from BLASTx (depicted in Fig. 2) could not be confirmed in the subsequent in silico analysis and were considered as possible artefacts from the NGS run (e.g., *Herpesvirales* and *Bamfordvirae*) as commonly described^30^. An overview of the confirmed results after quality checks is given in Table 1, specifying details of the obtained NGS data, related viruses and novel identified virus strains.

**Table 1:** Overview of the results obtained from mNGS data analysis of feces and urine swab samples from *M.* *phillipsi* bats collected in July 2018.

*Astroviridae*		
Name of related virus	**Bat astrovirus 1 isolate AFCD77 (EU847151)**	
Sample type	Feces samples	Urine swab samples
Pool numbers	F1, F2, F3, F4, F5, F6, F7, F8	U4, U8, U9, U14, U15
Assigned reads/longest contig	289/1068 nt	422/379 nt
Nucleotide identity	82%	85.6%
Name of novel virus strain	Bat astrovirus strain F2/18 (Acc. OP141159)	Bat astrovirus strain US2/18 (Acc. OP141166)
Name of related virus	**Mamastrovirus 14 isolate AFCD57 (NC_043099)**	
Sample type	Feces samples	
Pool numbers	F3	
Assigned reads/longest contig	1045/1366 nt	
Nucleotide identity	86.5%	
Name of novel virus strain	Mamastrovirus 14 strain F2/18 (Acc. OP141160)	
Name of related virus	**Mamastrovirus 18 isolate AFCD337 (NC_043102)**	
Sample type	Urine swab samples	
Pool numbers	U3, U5, U6, U9, U11, U12, U14	
Assigned reads/longest contig	282/311 nt	
Nucleotide identity	84.4%	
Name of novel virus strain	Mamastrovirus 18 strain US2/18 (Acc. OP141167)	
*Coronaviridae*		
Name of related virus	**Bat alphacoronavirus isolate batCoV/MinFul/2018/SriLanka (OL956935)**	
Sample type	Feces samples	Urine swab samples
Pool numbers	F1, F2, F3, F4, F5, F6, F7, F8	U2, U3, U4, U5, U6, U8, U9, U12, U13, U14
Assigned reads/longest contig	994/729 nt	10,753/1226 nt
Nucleotide identity	83.1%	98.6%
Name of novel virus strain	Bat alphacoronavirus strain MinPhil/F2/2018/SriLanka (Acc. OP141161)	Bat alphacoronavirus strain MinPhil/U2/2018/SriLanka (Acc. OP141168)
Name of related virus	**BtMf-AlphaCoV/GD2012 (KJ473797)**	
Sample type	Feces samples	Urine swab samples
Pool numbers	F1, F2, F3, F4, F5, F7	U4, U6, U9, U12, U13, U14
Assigned reads/longest contig	2443/1113 nt	2182/769 nt
Nucleotide identity	83.1%	85.5%
Name of novel virus strain	Bat alphacoronavirus strain AlphaCoV/F2/2018 (Acc. OP141162)	Bat alphacoronavirus strain AlphaCoV/U2/2018 (Acc. OP141169)
*Iflaviridae*		
Name of related virus	**Spodoptera exigua iflavirus 2 isolate Korean (JN870848)**	
Sample type	Feces samples	
Pool numbers	F1	
Assigned reads/longest contig	373/1283 nt	
Nucleotide identity	96.8%	
Name of novel virus strain	Spodoptera exigua iflavirus strain F2/18 (Acc. OP141163)	
*Paramyxoviridae*		
Name of related virus	**Jingmen Miniopterus schreibersii paramyxovirus 1 (MZ328288)**	
Sample type	Urine swab samples	
Pool numbers	U2, U3, U6, U7, U9, U11, U12, U14	
Assigned reads/longest contig	573/450 nt	
Nucleotide identity	84%	
Name of novel virus strain	Miniopterus phillipsi paramyxovirus strain US2/18 (Acc. OP141170)	
*Picornaviridae*		
Name of related virus	**Miniopterus schreibersii picornavirus 1 (JQ814851)**	
Sample type	Feces samples	
Pool numbers	F1, F2, F3, F4, F5, F6, F7, F8	
Assigned reads/longest contig	5714/3452 nt	
Nucleotide identity	86%	
Name of novel virus strain	Miniopterus phillipsi picornavirus strain F2/18 (Acc. OP141164)	
Unclassified *Riboviria*		
Name of related virus	**Hubei sobemo-like virus 21 strain CC64469 (KX882813)**	
Sample type	Feces samples	
Pool numbers	F1, F3, F4	
Assigned reads/longest contig	439/534 nt	
Nucleotide identity	82.1%	
Name of novel virus strain	Hubei sobemo-like virus strain F2/18 (Acc. OP141165)	

The table indicates the related viruses (in bold), sample types and pool numbers from which the results were obtained, including the number of assigned reads, longest contig and nucleotide identity. The name and accession number of the novel virus strains as uploaded to GenBank are indicated, respectively.

For the families *Coronaviridae*, *Picornaviridae*, *Astroviridae* and *Paramyxoviridae*, the presence of viral sequences was furthermore confirmed in the original RNA pools (see Table 1) by using PCR and specifically designed primers based on the NGS data. The remaining viruses (*Iflaviridae* and unclassified *Riboviria*) were solely confirmed with in silico analysis of the data by BLASTn.

From all viral assemblies, either the longest contig or the contig used for phylogenetic reconstruction was uploaded to GenBank. These sequences of newly detected virus strains were named in relation to the closest related reference virus and with respect to current ICTV classification criteria (see Table 1).

### *Astroviridae*

Astroviruses (n = 3) were found in F and US samples. In F samples, 1045 reads were assigned to Mamastrovirus 14 isolate AFCD57 (NC_043099) with a nucleotide identity of 86.8% on the longest contig (1366 nt), located overlapping on polyprotein 1AB and capsid protein CDS. Further 289 reads from F samples were assembled and mapped to Bat astrovirus 1 isolate AFCD77 polyprotein 1AB gene (EU847151) with a nucleotide identity of 82% on the longest contig (1068 nt).

A total of 422 reads from US samples were also assembled to EU847151, with a nucleotide identity of 85.6% on the longest contig (379 nt).

From US samples, further 282 reads were assembled to Mamastrovirus 18 isolate AFCD337 (NC_043102) with a nucleotide identity of 84.4% on the longest contig (311 nt), located on the polyprotein 1AB gene.

**Figure 3:**
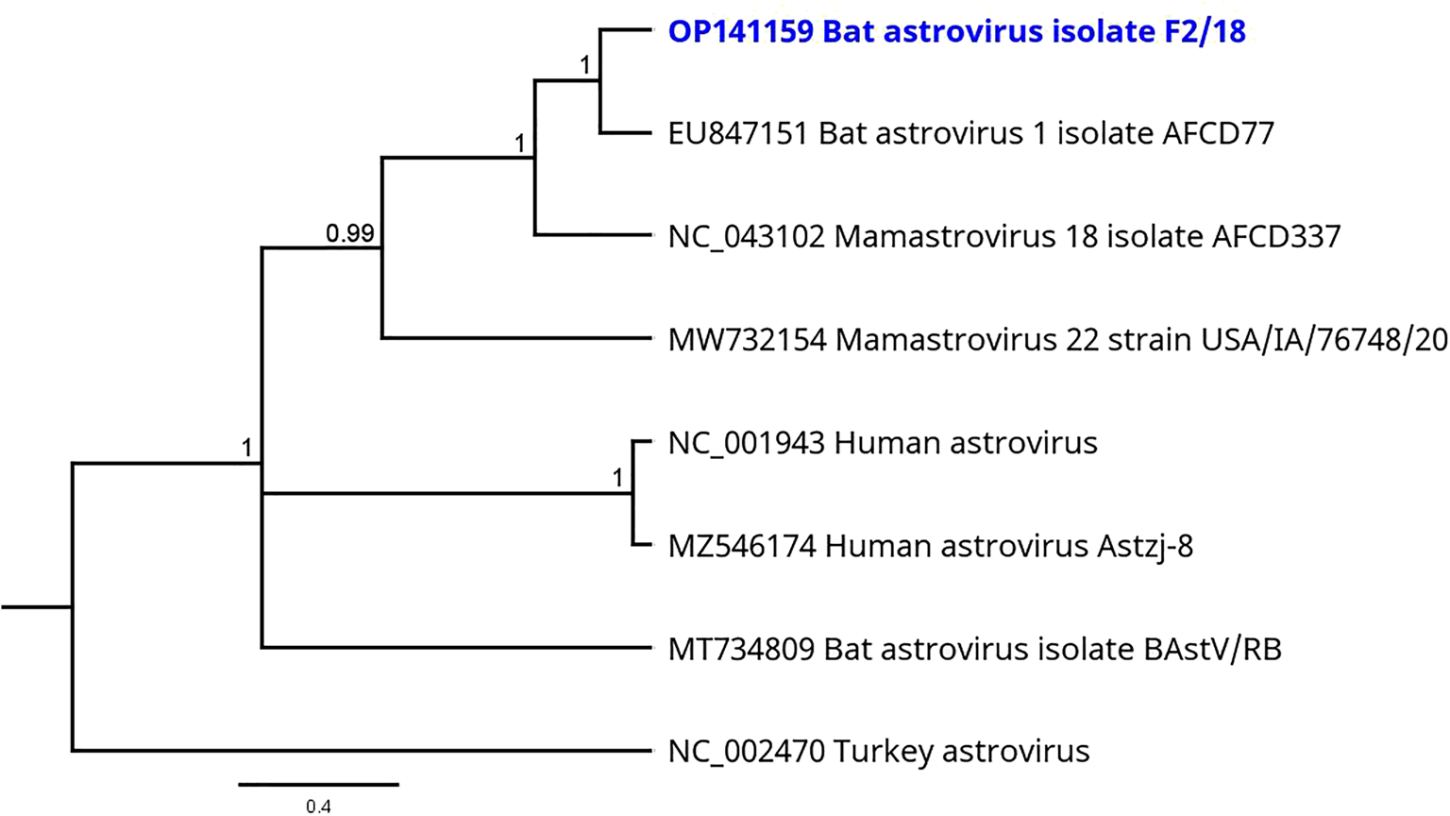
Phylogenetic tree based on the mNGS sequences obtained from the virome of F sample pools (highlighted in blue) and a selection of other astrovirus species. Turkey astrovirus (NC_002470) was used as outgroup. Phylogenetic reconstruction was calculated with the Bayesian MCMC algorithm: 500,000 generations were calculated with a subsampling frequency of 100 and a burn-in of 10%. The substitution model GTR was selected with a gamma-distributed rate variation. Visualized as molecular clock with uniform branch lengths.

Because of the lack of overlapping sequences, phylogenetic reconstruction was calculated exemplarily with Bat astrovirus strain F2/18 (OP141159); the results are shown in Fig. 3.

### *Coronaviridae*

**Figure 4:**
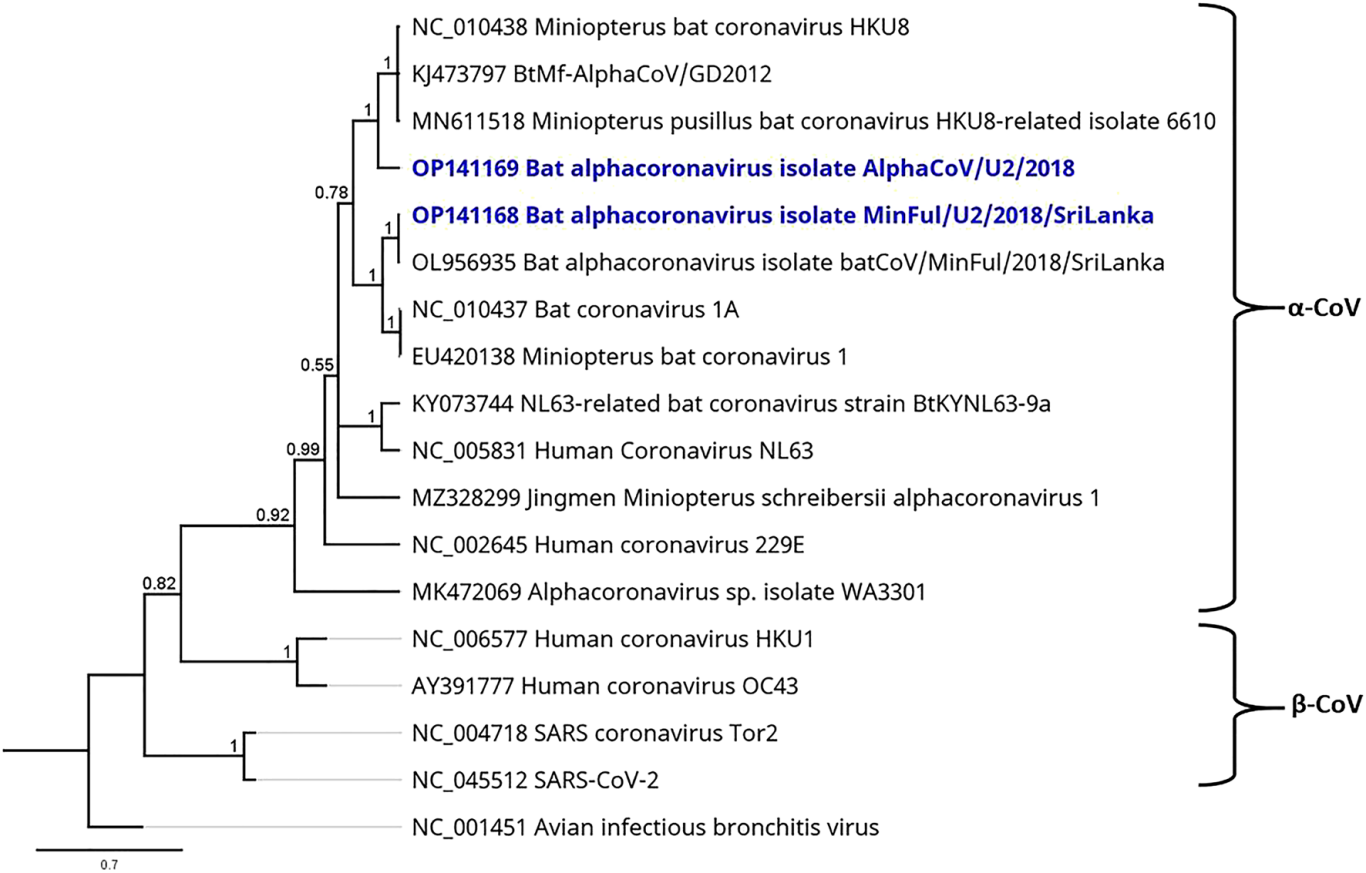
Phylogenetic tree based on a 224-nt contig on the conserved ORF1B CDS of CoV obtained from the virome of US sample pools (highlighted in blue) and a selection of α-CoVs and β-CoVs as specified. For use as outgroup, the γ-CoV avian infectious bronchitis virus (NC_001451) was included in the calculation. Phylogenetic reconstruction was calculated with the Bayesian MCMC algorithm: 1,000,000 generations were calculated with a subsampling frequency of 100 and a burn-in of 10%. The substitution model GTR was selected with a gamma-distributed rate variation. Visualized as molecular clock with uniform branch lengths.

Two coronaviruses (CoV) were found in US and F samples, respectively. A total of 994 reads from F samples and 10,753 reads from US samples were mapped to the bat alphacoronavirus strain batCoV/MinFul/2018/SriLanka (OL956935) with nt identities on conserved ORF1B CDS of 80.36% (F, 668 nt contig) and 98.37% (US, 735 nt contig). In addition, 2443 reads from F sample pools and 2182 reads from US sample pools were mapped to BtMf-AlphaCoV/GD2012 (KJ473797), an alphacoronavirus HKU8 strain from China. Nucleotide identities on the conserved ORF1B CDS were calculated with 82.9% (F, 1113 nt contig) and 85.5% (US, 769 nt contig). For phylogenetic reconstruction, contigs on the ORF1B CDS were selected. Because of the lack of overlapping contigs, phylogeny was calculated separately for the sequences from F and US samples but by using the same reference strains including common human pathogenic CoVs. For the conserved ORF1B CDS, overlapping contigs of 182 nt (F samples) and 224 nt (US samples) were obtained. Figure 4 shows the phylogenetic reconstruction of CoV from US sample pools.

The calculation confirms the presence of two different strains that are allocated to different branches inside the genus *Minunacovirus*. The identified Bat alphacoronavirus batCoV/MinFul/2018/SriLanka US2/2018 clusters with strains of the species Miniopterus bat coronavirus 1, whereas the other Miniopterus AlphaCoV strain US2/2018 clusters with HKU8-related strains. BatCoV/MinFul/2018/SriLanka US2/2018 was identified in the described sample set and further investigation, extension and analysis led to the assembly of the whole genome of this novel strain^20^.

### *Iflaviridae*

An iflavirus was found in F samples with a total of 373 reads assembled to Spodoptera exigua iflavirus 2 isolate Korean (JN870848), covering 93.6% of the genome. The longest contig of 1283 nt, located at the beginning of the polyprotein CDS, shares a nucleotide identity of 96.8% to the related Spodoptera exigua iflavirus 2.

### *Paramyxoviridae*

**Figure 5:**
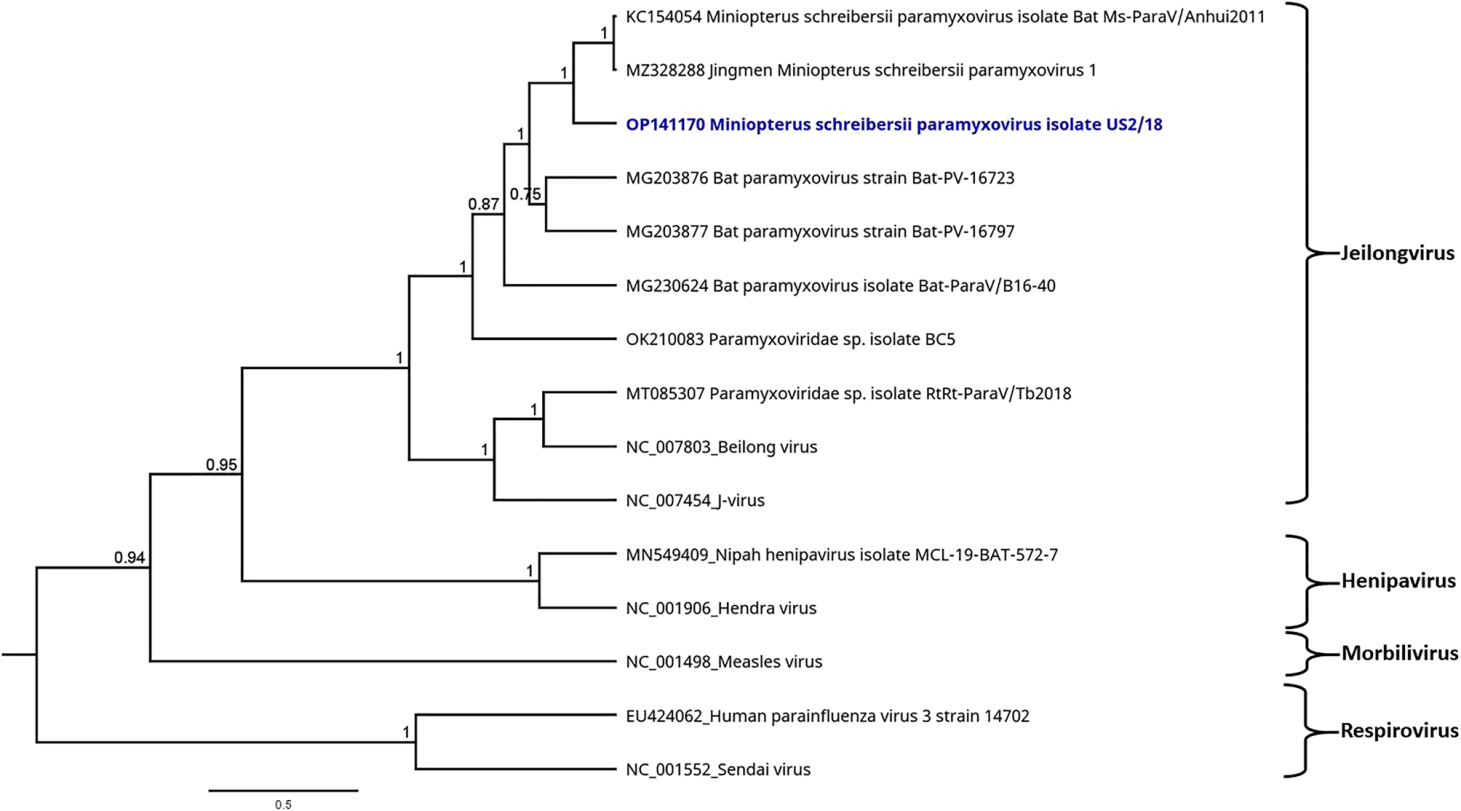
Phylogenetic tree based on the mNGS sequences obtained from the virome of US sample pools (highlighted in blue) and a selection of PMVs. Sendai virus (NC_001552) was selected as outgroup for the calculation. Phylogenetic reconstruction was calculated with the Bayesian MCMC algorithm: 1,000,000 generations were calculated with a subsampling frequency of 100 and a burn-in of 10%. The substitution model GTR was selected with a gamma-distributed rate variation. Visualized as molecular clock with uniform branch lengths.

A paramyxovirus (PMV) was found in US samples and confirmed by PCR (compare Table 1). A total of 573 reads were mapped to the full genome of Jingmen Miniopterus schreibersii paramyxovirus 1 (MZ328288). The longest contig (450 nt) on the conserved L gene showed highest nucleotide identities to this strain (84%) and to the partial genome of Miniopterus schreibersii paramyxovirus isolate Bat Ms-ParaV/Anhui2011 (KC154054; 84.6% nt identity). This contig was also selected for phylogenetic reconstruction. The phylogenetic tree of 16 paramyxoviruses, including the novel sequence from Sri Lanka and selected human pathogenic strains, is shown in Fig. 5.

The phylogenetic reconstruction confirms that the novel paramyxovirus from Sri Lanka is closely related to the two PMV strains as described before. Other Miniopterus-related PMVs from China cluster in the same branch of the tree, representing the subgenus Jeilong virus. Apart from this, the subgenera Henipavirus, Morbillivirus and Respirovirus were each allocated to distinct branches of the tree. These subgenera also include the human pathogenic strains that were selected for this phylogenetic reconstruction.

### *Picornaviridae*

**Figure 6:**
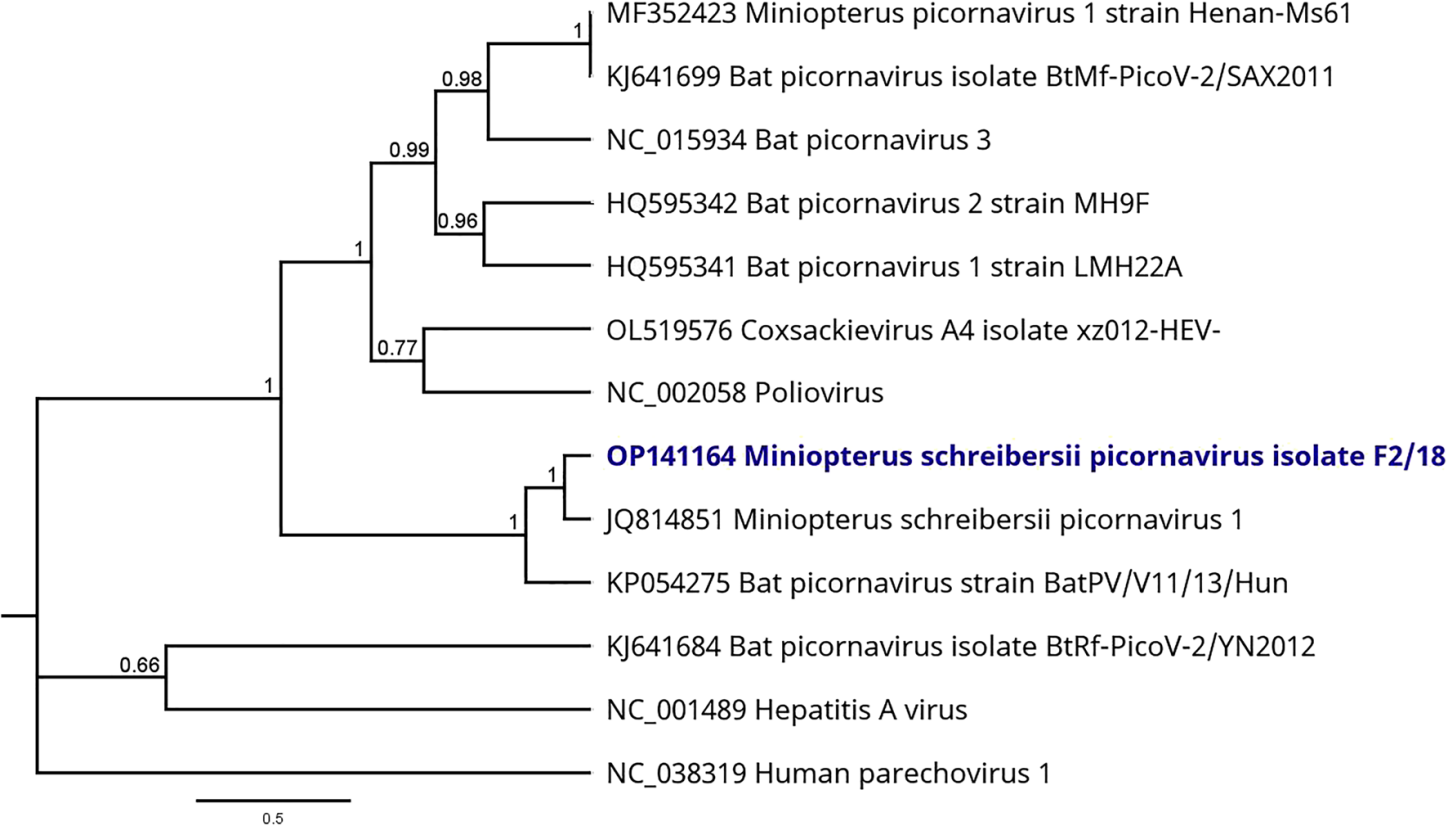
Phylogenetic tree based on the mNGS sequences obtained from the virome of F sample pools (highlighted in blue) and a selection of picornaviruses. Human parechovirus 1 (NC_038319) was selected as outgroup for the calculation. Phylogenetic reconstruction was calculated with the Bayesian MCMC algorithm: 1,000,000 generations were calculated with a subsampling frequency of 100 and a burn-in of 10%. The substitution model GTR was selected with a gamma-distributed rate variation. Visualized as molecular clock with uniform branch lengths.

A picornavirus was found in F samples: a total of 5714 NGS reads were assembled to Miniopterus schreibersii picornavirus 1 (JQ814851), covering most parts of the genome. The longest contig of 3452 nt, located at the end of the polyprotein CDS, shares a nucleotide identity of 86% to the Miniopterus schreibersii picornavirus 1. For phylogenetic reconstruction, a well-covered contig of 311 nt was selected on the highly conserved 2C peptide on the picornaviral polyprotein. The phylogenetic reconstruction is illustrated in Fig. 6.

As shown in Fig. 6, the novel bat picornavirus strain from F samples clusters monophyletically with its closest related strain Miniopterus schreibersii picornavirus 1 and the Bat picornavirus strain BatPV/V11/13/Hun, both obtained from *Miniopterus schreibersii* bats. Other bat- and Miniopterus-hosted picornaviruses were allocated to different branches of the phylogenetic tree. The human pathogenic representative picornaviruses are clearly distant from the novel Sri Lankan strain.

### Unclassified *Riboviria*

From F samples, 439 reads were assembled to a Hubei sobemo-like virus 21 strain CC64469 (KX882813) with a nucleotide identity of 82.1% on the longest contig (534 nt).

### Phages

A number of phages were found in each sample type as described. Considerable amounts of reads were assigned to the bacteriophage orders *Caudovirales* (OS: 576; F: 1278; US: 663,683) and *Petitvirales* (OS: 44,741; F: 235; US: 554,119), respectively.

## Discussion

In our study, we analyzed the virome of *M.* *phillipsi* bats inhabiting the Wavul Galge cave in the interior of the island of Sri Lanka. By taking oral swabs, urine swabs and feces, we aimed to examine if different viruses are detectable via differing shedding routes represented by the individual sample types. This assumption was confirmed and we have been able to obtain different virome compositions for each sample type.

In OS samples, the primary viral hits were assigned to phages belonging to the orders *Caudovirales* and *Petitvirales*. The presence of any other viral sequences in OS samples was not confirmed after quality assessment (in-depth analysis of sequences found and comparison with the database) of analyzed NGS data. However, the detection of *Caudovirales* and *Petitvirales* in OS underlines the possibility of virus detection also for this sample type.

Apart from this, different other viruses were detected in the F and US samples as discussed in the following sections.

### Phages and diet-related viruses

Sequence reads assigned to phages were found in high numbers in all three sample types. Although these were not inspected in detail in this study, the large number and variety of reads also indicates the presence of numerous bacteria in the collected samples. In combination with 16S bacterial metagenomic analysis, these results will be interesting to be further explored in future studies.

In F samples, viral sequences were identified matching Hubei sobemo-like virus 21 strain CC64469 (China), belonging to unclassified *Riboviria*. This virus was originally isolated from invertebrates within the phylum *Annelida*^31^. Most probably a worm carrying this virus was taken in as nutrition by the bat, passaged and excreted with the feces afterwards.

In addition to this, viral sequences assigned to Spodoptera exigua iflavirus 2 (Korea) were identified in F samples. This virus belonging to the family *Iflaviridae* within the order *Picornavirales* was isolated from insects of the genus *Spodoptera* (moths)^32^. Here again it is very likely that the virus was taken in with moths for nutrition, passaged and excreted afterwards. In both examples, it is impossible to conclude whether the virus infected the bats as well or was merely digested, passaged and excreted. For this purpose, tissues and organs would need to be investigated.

However, the identified phages as well as these two examples show that the viromes derived from NGS data are complex and do not only comprise the bat-related viruses. Moreover, the data also reveal a number of other viruses derived from the inherent bacterial flora within the bat as well as viruses derived from insects serving as nutrition for the bats. This in turn has the potential to analyze virome compositions of whole habitats, including different organisms. This may be the basis or part of further investigations regarding the bacterial flora and dietary habits of the bats.

### *Astroviridae*

The viruses within the family *Astroviridae* are common in a wide range of birds (genus *Avastroviruses*) and mammals (genus *Mamastrovirus*), including bats and humans^33^. Human astrovirus infections mainly cause gastroenteritis in children^34^. Members of this family have a high genetic diversity depending on the respective host species and a wide host range (birds and mammals). However, the zoonotic potential of bat-hosted astroviruses is widely unexplored as yet^35^. Members of the *Mamastrovirus* genus are classified based on the amino acid sequences of the capsid region and can be further divided into species, depending on the host and other genetic criteria^36^. Based on the analyzed sequence data, the examined bats carried multiple astrovirus strains. Thus, bat astrovirus sequences were detected for the first time in a bat species from Sri Lanka, namely *M.* *phillipsi*.

From US samples, Bat astrovirus strain US2/18 (OP141166) and Mamastrovirus 18 strain US2/18 (OP141167) were identified, whereas Bat astrovirus isolate F2/18 (OP141159) and Mamastrovirus 14 strain F2/18 (OP141160) were identified from F samples. All three viruses were originally detected in *Miniopterus* bat species, which may indicate a high host specificity of these viruses. In addition, the circulation of different bat astrovirus strains within one bat population is conceivable, as already reported in other studies^37–39^.

Phylogenetic reconstruction allowed for a rough classification of the Bat astrovirus strain F2/18 from Sri Lanka, as an example. Although only few suitable reference strains from bats were available in the databases, the phylogeny distinguished astroviruses of bats from astroviruses of other vertebrates (turkey or human) by allocation to different tree branches.

Most available bat astroviral references from the databases contained partial genomes only, located in different gene regions. Further sequence analysis and phylogenetic comparison between all newly obtained astrovirus sequences from Sri Lanka were therefore not possible with the available data. Consequently, we cannot finally prove the presence of multiple astrovirus strains in the collected samples. It may be possible that the sequences were originally derived from a single astrovirus but were mapped to the different partial genomes obtained from the database. Therefore isolation of these viruses followed by in-depth sequencing of the missing genome sequences could be helpful in order to obtain full genome data or at least full gene sequences. With further sequence information it could be examined whether the astroviral reads were actually derived from different strains or whether they belonged to a single astrovirus within the samples.

### *Coronaviridae*

Presence of two different CoVs in *M.* *phillipsi* bats was confirmed in F and US samples. Both related virus strains were originally detected in *Miniopterus* bats^20,40,41^. The CoV full genome from Sri Lanka (OL956935) was derived from rectal swabs collected during the same bat sampling session as this study and was reported previously^20^. With the virome sequence data of F and US samples in this study, we were able to confirm the presence of this virus strain also in these samples. In addition, we identified Bat alphacoronavirus strain AlphaCoV/F2/2018 and Bat alphacoronavirus strain AlphaCoV/U2/2018 which are closely related to Miniopterus bat coronavirus HKU8 strains. All identified viruses belong to *Minunacoviruses*, an α-CoV subgenus containing *Miniopterus*-hosted viruses. The slight differences between the two virus species within the subgenus are also visible in the phylogenetic reconstruction of different CoVs. Representative strains of the virus species Miniopterus bat coronavirus 1 and Miniopterus bat coronavirus HKU8 were divided into two different clusters within the branch of *Minunacoviruses*. Co-existence of these two virus species and different CoVs in general have already been reported before^42–44^. Therefore it can be assumed that these two or even more CoV strains circulate steadily in this population of *M.* *phillipsi* bats. Regarding the zoonotic potential of the detected CoVs, further investigation of full genes and specific receptor-binding domains would be necessary for more precise statements. Based on the available data and phylogenies calculated from this, we did not find indications for a human pathogenic potential of the detected viruses^17,20^. Human pathogenic CoVs that cause outbreaks and pandemics, like SARS, MERS and Covid-19, all belong to the genus of β-CoV and are genetically diverse from the genus α-CoV. Although mildly human pathogenic viruses such as HCoVs NL63 and 229E are represented in the α-CoV genus as well, the phylogeny ranks these species as rather distantly related to the *Minunacoviruses*.

### *Paramyxoviridae*

Representatives of *Chiroptera*-hosted PMVs are able to cause zoonotic diseases in humans; therefore this virus family was of high interest for the virome analysis of the bat samples^45^. The detection of PMVs in US samples was expected, as this is the usual shedding route of these viruses^46^. The presence of PMVs in the collected US samples has been reported before by using semi-nested PCR^7^ and Sanger sequencing^18^. These results indicated the presence of multiple PMVs in the examined samples. The co-circulation of multiple PMVs in general is common in bats and has been reported before^47,48^. In this study, we proved the presence of previously identified PMVs in the samples by using another molecular virus detection method. However, the NGS data did not reveal enough sequence information to confirm multiple PMV strains. For this purpose, NGS of single US samples instead of pools or more sequencing depth would be necessary to compare sequence data between individuals.

As expected, the identified Miniopterus schreibersii paramyxovirus strain US2/18 (OP141170) has the highest similarities to other *Miniopterus*-hosted PMVs. In the phylogenetic tree, all *Miniopterus*-derived strains are assigned to the group of Jeilong virus (Fig. 5). The other branches of the phylogenetic tree depict only a small number of representative strains, including human pathogenic PMVs, whereas the actual *Orthoparamyxovirinae* subfamily is notably more diverse^45,49^. In accordance to this, different PMVs can cause diseases of different severity in humans (e.g., Human parainfluenzavirus vs. Nipah virus), whereas other PMVs have a rather low zoonotic potential. The preliminary analysis of the limited sequence data and the phylogenetic reconstruction give no indication of a human pathogenic potential of the novel PMVs. Further sequence data will be needed to allow for a detailed and complex analysis and taxonomic classification of the novel PMV strain^50^.

### *Picornaviridae*

The family of picornaviruses is a highly diverse family with 68 genera and 158 virus species according to ICTV^36^. They are globally distributed in a number of bat species including *Miniopterus* bats^51,52^. Additionally, they are found in a number of other host species including birds, livestock and humans. Cross-species transmissions between different bats or mammals are possible as well as zoonotic transmissions to humans^53^. In humans, picornaviruses such as enterovirus, rhinovirus, coxsackievirus, hepatovirus A and human parechovirus can cause diseases of the nervous system and the respiratory and gastrointestinal tracts^54^.

A number of sequences related to Miniopterus schreibersii picornavirus 1 were identified in F samples, which is a described shedding route of bat picornaviruses^55^. The novel strain from Sri Lanka, Miniopterus schreibersii picornavirus strain F2/18 (OP141164), shares an identity of 86% to the reference strain from China^56^. For phylogenetic reconstruction in this study, a suitable sequence on the 2C peptide was selected which is a highly conserved region on the picornaviral polyprotein and therefore suitable for this analysis^57^. The phylogenetic reconstruction included several bat picornaviruses and human pathogenic strains. The novel picornavirus strain from Sri Lankan *M.* *phillipsi* bats was assigned to a branch with other *Miniopterus*-hosted picornaviruses, and the human pathogenic species were assigned to other branches of the tree. The available results did not indicate a human pathogenic potential of the identified picornavirus. Although the phylogenetic analysis was limited to a small proportion of the *Picornaviridae* family, we were able to get a general idea on phylogenetic relationships of the novel bat picornavirus from Sri Lanka. For proper species classification, a full protein sequence analysis of P1, 2C, 3C and 3D proteins will be necessary but was not possible from the obtained data. However, the results represent the first detection of a picornavirus in the bat species *M.* *phillipsi* from Sri Lanka.

## Conclusion

We were able to analyze the different compositions of the *M.* *phillipsi* virome by potential shedding route obtained from oral swabs, urine swabs and feces samples. Depending on the sample types, different viruses were detected by using NGS analysis, each corresponding to their typical shedding routes.

Independent of the sample type, we were able to detect the co-existence of astroviruses, coronaviruses, paramyxoviruses and picornaviruses circulating simultaneously in the *M.* *phillipsi* bat population. However, we point out that viruses in excretions, i.e., urine and feces, can naturally cross-contaminate each other. We assess the possibility of detecting viruses by different sampling routes; the detection does not necessarily represent the origin of replication and the transmission route. Co-existence of these viruses may be common in bats, and even a co-speciation of virus species with their specific host is discussed^58,59^. It is assumed that virus–virus interaction is also possible and may influence the host, resulting in very specific viral shedding patterns depending on the virome composition^48^. In future, the epidemiological consequences of co-existing viruses in the bats should be further examined.

It is remarkable that mainly bacteriophages were identified in OS samples, although saliva is also known as common shedding route for other virus families. Lyssaviruses, including bat rabies-related ones, have been detected in other studies focusing on bat lyssaviruses; if these viruses are prevalent in the bats they are excreted by salivary glands and therefore shed with the saliva^60^. However, the excretion of viruses is generally affected by seasonal patterns and may have been low at the respective sampling point. Since we only used non-invasive sampling methods and could not examine bat brain or other tissue samples, we cannot conclude whether or not such viruses were prevalent in bat organs at the time of bat sampling. A long-term and frequent bat sampling will help to understand seasonality and shedding patterns of different viruses of interest. Probably the virome of all different sample types would change over time as it is influenced by seasons and environmental factors like temperature, humidity, rainfall, migration and reproduction cycles^1,48,61,62^.

In general, all collected sample types also represent possible transmission routes from bats to humans. The way of viral shedding depends on the respective tissue where the virus replicates: e.g., replication in kidneys and shedding via urine or replication in intestine organs and shedding via feces^63^.

Transmission of viruses via saliva would be possible from bites when catching and handling the bats. Urine and feces are constantly shed by the bats in the Wavul Galge cave, and the intake of these aerosols containing viral particles may possibly lead to virus exposure when entering the cave without any protective equipment^64^. In this context, it would also be of interest in the future to investigate the virome of the other bat species inhabiting the cave and to determine whether they are also susceptible to the same viruses.

Although the fecal–oral transmission route is rather unlikely, local people are in close contact to bats when collecting bat guano to use as organic fertilizer^65^. Especially in rural areas like those around the Wavul Galge cave, the use of bat guano in agriculture is common and represents a potential transmission risk to the farmers. Although our data points towards a rather low zoonotic potential, this does not exclude the seasonal presence of potentially pathogenic agents. A special awareness regarding possible transmissions should be raised. Concurrently, the fear of zoonotic viruses in bat hosts should not justify their eradication. On the contrary, the natural habitat of the bat population in the cave should be recognized and respected.

In summary, the virome composition of different sample types obtained from *M.* *phillipsi* bats in Sri Lanka was analyzed for the first time. Recently, DNA barcoding and morphological studies on this species suggest that the *Miniopterus* bats inhabiting the island of Sri Lanka are in fact a new species of bat not described hitherto and named *Miniopterus phillipsi*^66^. All studies on *Miniopterus* bats from Sri Lanka, conducted before the renaming of the species, identified them as *Miniopterus fuliginosus* due to lack of more precise information in the database. Based on these findings, the results from our work would represent the first virome analysis for this newly described bat species. This study is the baseline for further in-depth investigation of pathogens of bats inhabiting the Wavul Galge cave in Sri Lanka.

### Supplementary Information

Supplementary Information.

## Data Availability

The datasets generated and analyzed during the current study are available in GenBank of the National Center for Biotechnology Information (NCBI) repository, accession numbers OP141159 to OP141169.
