# Supplementary material for: Comparative virome analysis of individual shedding routes of Miniopterus phillipsi bats inhabiting the Wavul Galge cave, Sri Lanka
Source: Sci Rep. 2023 Aug 8;13:12859. doi: 10.1038/s41598-023-39534-3 (PMC10409741; doi:10.1038/s41598-023-39534-3)
Supplement: Supplementary file 1 — Supplementary Information. [file 41598_2023_39534_MOESM1_ESM.docx]

***Comparative virome analysis of individual shedding routes of* Miniopterus phillipsi *bats inhabiting the Wavul Galge Cave, Sri Lanka***

**Therese Muzeniek ^1^, Thejanee Perera ^2^, Sahan Siriwardana ^3^, Dilara Bas ^1^, Fatimanur Bayram ^1^, Mizgin Öruc ^1^, Beate Becker-Ziaja ^4^, Inoka Perera ^3^, Jagathpriya Weerasena ^2^, Shiroma Handunnetti ^2^, Franziska Schwarz ^1^, Gayani Premawansa ^5^, Sunil Premawansa ^3^, Wipula Yapa ^3^, Andreas Nitsche ^1^ and Claudia Kohl ^1^***

^1^ Robert Koch Institute, Centre for Biological Threats and Special Pathogens, Highly Pathogenic Viruses (ZBS 1), 13353 Berlin, Germany

^2^ Institute of Biochemistry, Molecular Biology and Biotechnology, University of Colombo, 00300 Colombo, Sri Lanka

^3^ IDEA (Identification of Emerging Agents) Laboratory, Department of Zoology and Environment Sciences, University of Colombo, 00300 Colombo, Sri Lanka

^4^ Robert Koch Institute, Centre for International Health Protection, Public Health Laboratory Support (ZIG 4), 13353 Berlin, Germany

^5^ Colombo North Teaching Hospital, 11010 Ragama, Sri Lanka

***** Correspondence: KohlC@rki.de; Tel.: +49 30 187 542 144

Table ST1: Overview of the sampled *Miniopterus phillipsi* bats individuals, the age status and sex, collected sample types and their assignment to Pools for Next Generation Sequencing

| **Bat Number** | **Age status** | **Sex** | **Overview of collected samples, their assignment to NGS pools and the read output per pool** | | | | | | |
| --- | --- | --- | --- | --- | --- | --- | --- | --- | --- |
|  |  |  | **Oral Swab** | **OS Pool for NGS** | **Urine Swab** | **U Pool for NGS** | **Feces sample** | **F Pool for NGS** |  |
| 85 | Adult | M | X | O1 |  | U2 |  | F1 |  |
| 87 | Adult | F | X |  | X |  |  |  |  |
| 88 | Sub-Adult | M | X |  |  |  |  |  |  |
| 89 | Sub-Adult | F | X |  |  |  |  |  |  |
| 91 | Adult | F | X |  |  |  | X |  |  |
| 94 | Adult | M | X |  |  |  | X |  |  |
| 95 | Adult | F | X |  | X |  |  |  |  |
| 96 | Sub-Adult | F | X |  | X |  |  |  |  |
| 98 | Adult | F | X |  |  |  |  |  |  |
| 100 | Adult | M | X |  | X |  | X |  |  |
| 101 | Adult | F | X | O2 | X |  | X |  |  |
| 103 | Adult | F | X |  | X |  |  |  |  |
| 104 | Adult | F | X |  | X |  |  |  |  |
| 106 | Adult | F | X |  | X |  |  |  |  |
| 107 | Adult | F | X |  |  |  |  |  |  |
| 108 | Adult | F | X |  |  |  |  |  |  |
| 109 | Adult | M | X |  |  |  |  |  |  |
| 110 | Adult | F | X |  |  |  |  |  |  |
| 111 | Adult | F | X |  | X |  |  |  |  |
| 113 | Adult | F | X |  | X |  | X |  |  |
| 114 | Adult | F | X | O3 | X | U3 |  |  |  |
| 115 | Adult | F | X |  |  |  |  |  |  |
| 116 | Adult | F | X |  | X |  |  |  |  |
| 117 | Adult | F | X |  | X |  |  |  |  |
| 118 | Adult | F | X |  |  |  |  |  |  |
| 119 | Adult | F | X |  | X |  | X |  |  |
| 120 | Adult | F | X |  | X |  |  |  |  |
| 121 | Adult | F | X |  |  |  |  |  |  |
| 122 | Adult | F | X |  |  |  |  |  |  |
| 123 | Adult | F | X |  |  |  | X |  |  |
| 124 | Adult | F | X | O4 |  |  |  |  |  |
| 125 | Adult | F | X |  |  |  | X |  |  |
| 126 | Adult | F | X |  |  |  |  |  |  |
| 127 | Adult | F | X |  |  |  | X |  |  |
| 128 | Adult | M | X |  |  |  | X |  |  |
| 129 | Adult | F | X |  |  |  |  | F2 |  |
| 130 | Adult | F | X |  |  |  |  |  |  |
| 131 | Adult | F | X |  |  |  | X |  |  |
| 132 | Adult | F | X |  | X |  |  |  |  |
| 133 | Adult | M | X |  |  |  |  |  |  |
| 134 | Adult | F | X | O5 | X |  | X |  |  |
| 135 | Adult | F | X |  | X |  |  |  |  |
| 136 | Adult | F | X |  | X | U4 |  |  |  |
| 137 | Adult | F | X |  | X |  |  |  |  |
| 138 | Adult | F | X |  |  |  |  |  |  |
| 139 | Adult | M | X |  | X |  |  |  |  |
| 140 | Adult | F | X |  |  |  |  |  |  |
| 142 | Adult | F | X |  |  |  | X |  |  |
| 143 | Adult | F | X |  | X |  | X |  |  |
| 144 | Adult | F | X |  |  |  |  |  |  |
| 145 | Adult | F | X | O6 |  |  |  |  |  |
| 146 | Adult | F | X |  | X |  |  |  |  |
| 147 | Adult | F | X |  | X |  | X |  |  |
| 148 | Adult | F | X |  | X |  | X |  |  |
| 149 | Adult | F | X |  |  |  | X |  |  |
| 150 | Adult | F | X |  |  |  | X |  |  |
| 152 | Adult | F | X |  |  |  |  |  |  |
| 153 | Adult | M | X |  |  |  | X |  |  |
| 154 | Sub-Adult | F | X |  |  |  |  |  |  |
| 155 | Adult | F |  |  | X |  | X |  |  |
| 156 | Adult | F | X |  |  |  | X | F3 |  |
| 157 | Adult | F | X | O7 |  |  |  |  |  |
| 158 | Adult | F | X |  |  |  |  |  |  |
| 159 | Adult | F | X |  | X |  |  |  |  |
| 160 | Sub-Adult | F | X |  | X |  |  |  |  |
| 161 | Adult | F | X |  |  | U5 | X |  |  |
| 162 | Adult | F | X |  |  |  |  |  |  |
| 163 | Adult | F | X |  |  |  |  |  |  |
| 164 | Adult | F | X |  |  |  |  |  |  |
| 165 | Adult | M | X |  | X |  |  |  |  |
| 166 | Adult | F | X |  | X |  |  |  |  |
| 167 | Adult | F | X | O8 | X |  |  |  |  |
| 168 | Adult | F | X |  | X |  |  |  |  |
| 169 | Adult | F | X |  | X |  |  |  |  |
| 170 | Adult | F | X |  | X |  |  |  |  |
| 171 | Adult | M | X |  | X |  |  |  |  |
| 172 | Adult | F | X |  | X |  |  |  |  |
| 173 | Adult | F | X |  |  |  | X |  |  |
| 174 | Adult | F | X |  |  |  |  |  |  |
| 175 | Adult | F | X |  | X |  |  |  |  |
| 176 | Adult | F | X |  | X |  |  |  |  |
| 177 | Adult | F | X | O9 | X | U6 |  |  |  |
| 178 | Adult | F | X |  | X |  |  |  |  |
| 179 | Adult | F | X |  |  |  | X |  |  |
| 180 | Adult | F | X |  | X |  | X |  |  |
| 181 | Adult | F | X |  | X |  |  |  |  |
| 182 | Adult | F | X |  |  |  | X |  |  |
| 183 | Adult | M | X |  | X |  | X |  |  |
| 184 | Adult | M | X |  | X |  |  |  |  |
| 185 | Adult | F | X |  |  |  |  |  |  |
| 186 | Adult | F | X |  | X |  | X |  |  |
| 187 | Adult | F | X | O10 | X | U7 |  |  |  |
| 188 | Adult | F | X |  |  |  |  |  |  |
| 189 | Adult | F | X |  | X |  | X |  |  |
| 190 | Adult | M | X |  | X |  |  |  |  |
| 191 | Adult | F | X |  | X |  |  |  |  |
| 192 | Adult | M | X |  |  |  | X |  |  |
| 193 | Adult | F | X |  | X |  |  | F4 |  |
| 194 | Adult | F | X |  |  |  |  |  |  |
| 195 | Adult | F | X |  | X |  | X |  |  |
| 196 | Adult | F | X |  | X |  |  |  |  |
| 197 | Adult | F | X | O11 | X | U8 |  |  |  |
| 198 | Adult | F | X |  | X |  |  |  |  |
| 199 | Adult | F | X |  | X |  |  |  |  |
| 200 | Adult | F | X |  |  |  | X |  |  |
| 201 | Adult | M | X |  | X |  |  |  |  |
| 202 | Adult | M | X |  | X |  | X |  |  |
| 203 | Adult | F | X |  | X |  | X |  |  |
| 204 | Adult | F | X |  | X |  | X |  |  |
| 205 | Adult | F | X |  | X |  |  |  |  |
| 206 | Adult | F | X |  | X |  |  |  |  |
| 207 | Adult | M | X | O12 | X | U9 |  |  |  |
| 208 | Adult | F | X |  |  |  | X |  |  |
| 209 | Adult | F | X |  | X |  | X |  |  |
| 210 | Adult | F | X |  |  |  |  |  |  |
| 211 | Adult | F | X |  |  |  |  |  |  |
| 212 | Adult | M | X |  |  |  |  |  |  |
| 213 | Adult | F | X |  |  |  | X |  |  |
| 214 | Adult | F | X |  |  |  |  |  |  |
| 215 | Adult | F | X |  |  |  | X |  |  |
| 216 | Adult | F | X |  |  |  |  |  |  |
| 217 | Adult | F | X | O13 | X |  |  | F5 |  |
| 218 | Adult | M | X |  | X |  | X |  |  |
| 219 | Adult | M | X |  |  |  |  |  |  |
| 220 | Adult | F | X |  |  |  | X |  |  |
| 221 | Adult | F | X |  |  |  | X |  |  |
| 222 | Adult | F | X |  |  |  | X |  |  |
| 223 | Adult | F | X |  |  |  |  |  |  |
| 224 | Adult | F | X |  |  |  |  |  |  |
| 225 | Adult | F | X |  | X |  |  |  |  |
| 226 | Adult | F | X |  |  |  | X |  |  |
| 227 | Adult | F | X | O14 | X | U10 | X |  |  |
| 228 | Adult | F | X |  |  |  | X |  |  |
| 229 | Adult | F | X |  | X |  |  |  |  |
| 230 | Adult | F | X |  | X |  | X |  |  |
| 231 | Adult | M | X |  |  |  |  |  |  |
| 232 | Adult | F | X |  | X |  |  |  |  |
| 233 | Adult | F | X |  |  |  | X |  |  |
| 234 | Adult | F | X |  | X |  | X |  |  |
| 235 | Adult | F | X |  |  |  | X | F6 |  |
| 236 | Adult | F | X |  | X |  | X |  |  |
| 237 | Adult | F | X | O15 |  | U11 | X |  |  |
| 238 | Adult | F | X |  |  |  | X |  |  |
| 239 | Adult | F | X |  | X |  | X |  |  |
| 240 | Adult | F | X |  | X |  |  |  |  |
| 241 | Adult | F | X |  | X |  | X |  |  |
| 242 | Adult | F | X |  | X |  | X |  |  |
| 243 | Adult | F | X |  |  |  | X |  |  |
| 244 | Adult | F | X |  | X |  | X |  |  |
| 245 | Adult | F | X |  |  |  | X |  |  |
| 246 | Adult | F | X |  | X |  | X | F7 |  |
| 247 | Adult | F | X | O16 | X | U12 | X |  |  |
| 248 | Adult | F | X |  | X |  |  |  |  |
| 249 | Adult | F | X |  | X |  | X |  |  |
| 250 | Adult | F | X |  |  |  | X |  |  |
| 251 | Adult | F | X |  |  |  | X |  |  |
| 252 | Adult | F | X |  |  |  | X |  |  |
| 253 | Adult | F | X |  | X |  |  |  |  |
| 254 | Adult | F | X |  | X |  | X |  |  |
| 255 | Adult | F | X |  | X |  |  |  |  |
| 256 | Adult | M | X |  | X |  | X |  |  |
| 257 | Adult | F | X | O17 |  | U13 |  |  |  |
| 258 | Adult | F | X |  | X |  | X |  |  |
| 259 | Adult | F | X |  |  |  | X |  |  |
| 260 | Adult | F | X |  | X |  |  | F8 |  |
| 261 | Adult | F | X |  | X |  | X |  |  |
| 262 | Adult | F | X |  | X |  |  |  |  |
| 263 | Adult | F | X |  | X |  | X |  |  |
| 264 | Adult | F | X |  | X |  |  |  |  |
| 265 | Adult | F | X |  | X |  |  |  |  |
| 266 | Adult | F | X |  | X |  |  |  |  |
| 267 | Adult | F | X | O18 | X | U14 |  |  |  |
| 268 | Adult | F | X |  | X |  | X |  |  |
| 270 | Adult | F | X |  | X |  | X |  |  |
| 271 | Adult | F | X |  | X |  |  |  |  |
| 272 | Adult | F | X |  | X |  |  |  |  |
| 273 | Adult | F | X |  | X |  |  |  |  |
| 274 | Adult | M | X |  |  |  |  |  |  |
| 275 | Adult | F | X |  |  |  |  |  |  |
| 276 | Adult | F | X |  |  |  |  |  |  |
| 277 | Adult | M | X | O19 |  |  |  |  |  |
| 278 | Adult | F | X |  | X |  |  |  |  |
| 279 | Adult | F | X |  |  |  | X |  |  |
| 280 | Adult | F | X |  |  |  | X |  |  |
|  |  |  |  |  |  |  |  |  |  |
| 281 | Adult | F | X |  | X |  |  |  |  |
| 282 | Adult | F | X |  |  |  | X |  |  |
| 283 | Adult | F | X |  |  |  | X |  |  |
| 284 | Adult | F | X |  | X |  |  |  |  |

Table ST2: Overview of the sample pools for NGS, obtained reads after trimming and total reads that were assigned to viruses in MEGAN after DIAMOND BLASTx.

| **Sample type** | **Pool** | **Total reads after trimming** | **Total reads assigned to viruses (MEGAN)** |
| --- | --- | --- | --- |
| **Oral swabs** | O1 | 4,906,956 | 3,005 |
|  | O2 | 5,381,629 | 1,757 |
|  | O3 | 5,416,413 | 3,317 |
|  | O4 | 4,568,104 | 1,732 |
|  | O5 | 4,609,884 | 1,776 |
|  | O6 | 6,206,508 | 2,584 |
|  | O7 | 4,984,406 | 2,731 |
|  | O8 | 4,570,809 | 1,153 |
|  | O9 | 3,241,858 | 2,412 |
|  | O10 | 4,515,414 | 1,629 |
|  | O11 | 3,963,803 | 3,142 |
|  | O12 | 3,794,828 | 1,578 |
|  | O13 | 4,025,694 | 1,881 |
|  | O14 | 4,549,502 | 2,165 |
|  | O15 | 3,726,854 | 2,247 |
|  | O16 | 5,625,662 | 1,465 |
|  | O17 | 8,428,422 | 4,434 |
|  | O18 | 6,145,989 | 1,769 |
|  | O19 | 3,905,991 | 2,243 |
| **Urine swabs** | U2 | 5,772,514 | 19,022 |
|  | U3 | 4,231,010 | 99,135 |
|  | U4 | 3,626,749 | 80,589 |
|  | U5 | 5,557,808 | 113,844 |
|  | U6 | 6,366,695 | 133,523 |
|  | U7 | 9,849,006 | 209,126 |
|  | U8 | 9,274,541 | 200,733 |
|  | U9 | 8,084,957 | 252,039 |
|  | U10 | 4,264,597 | 90,724 |
|  | U11 | 3,652,926 | 76,205 |
|  | U12 | 4,937,283 | 11,959 |
|  | U13 | 3,537,353 | 71,699 |
|  | U14 | 922,687 | 17,963 |
| **Feces** | F1 | 2,847,745 | 4,079 |
|  | F2 | 2,937,757 | 2,532 |
|  | F3 | 3,454,655 | 15,268 |
|  | F4 | 2,404,303 | 3,173 |
|  | F5 | 2,921,352 | 2,426 |
|  | F6 | 1,497,502 | 882 |
|  | F7 | 2,564,216 | 749 |
|  | F8 | 2,993,081 | 898 |


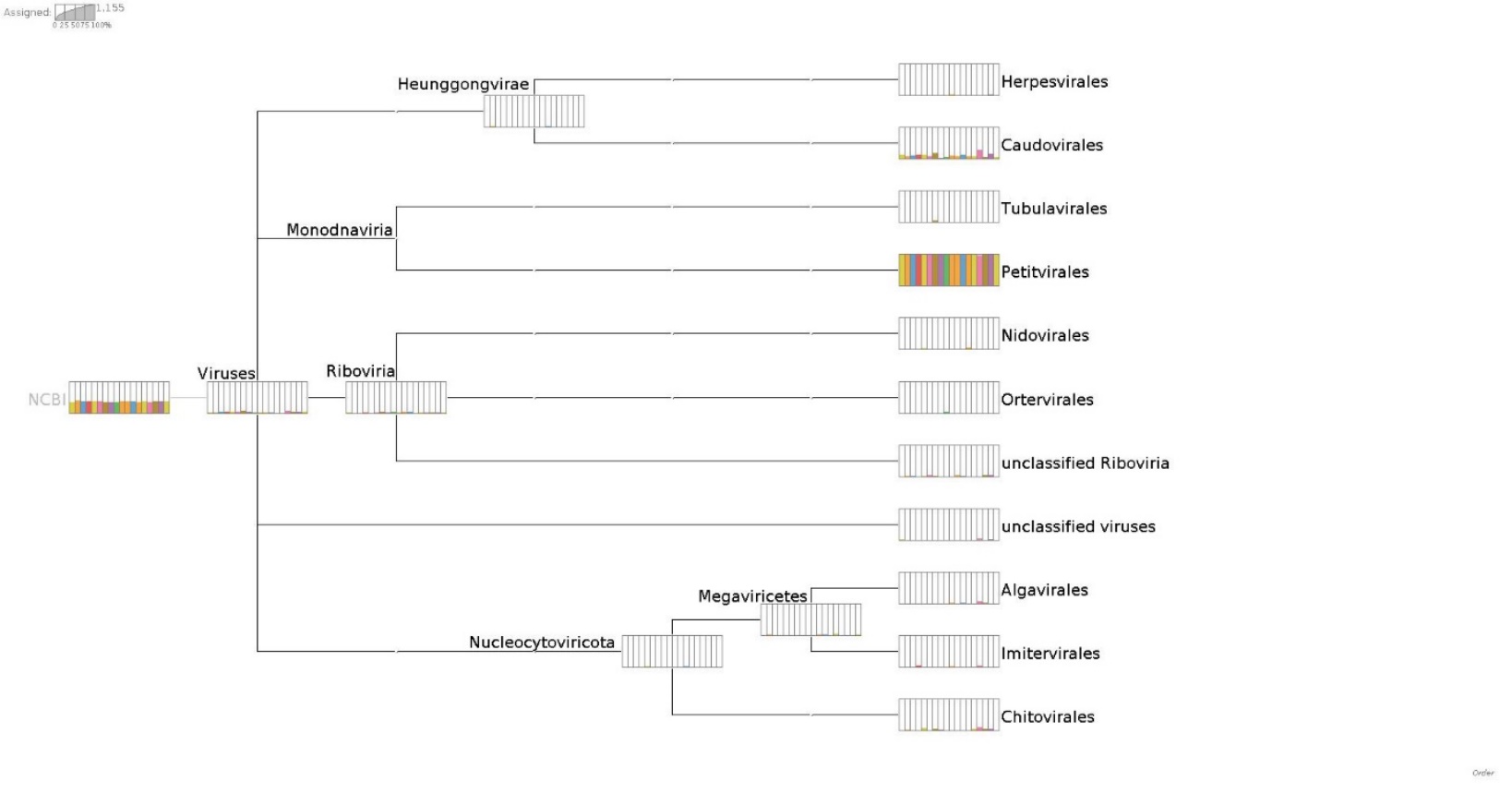


*Figure SF1: Normalized comparison of viral hits obtained after mNGS from OS pools OS2.1 – OS2.19, analyzed with diamond BLASTx algorithm and visualized in MEGAN software.* *Pools are displayed from OS2.1-OS2.19 from left to right.*


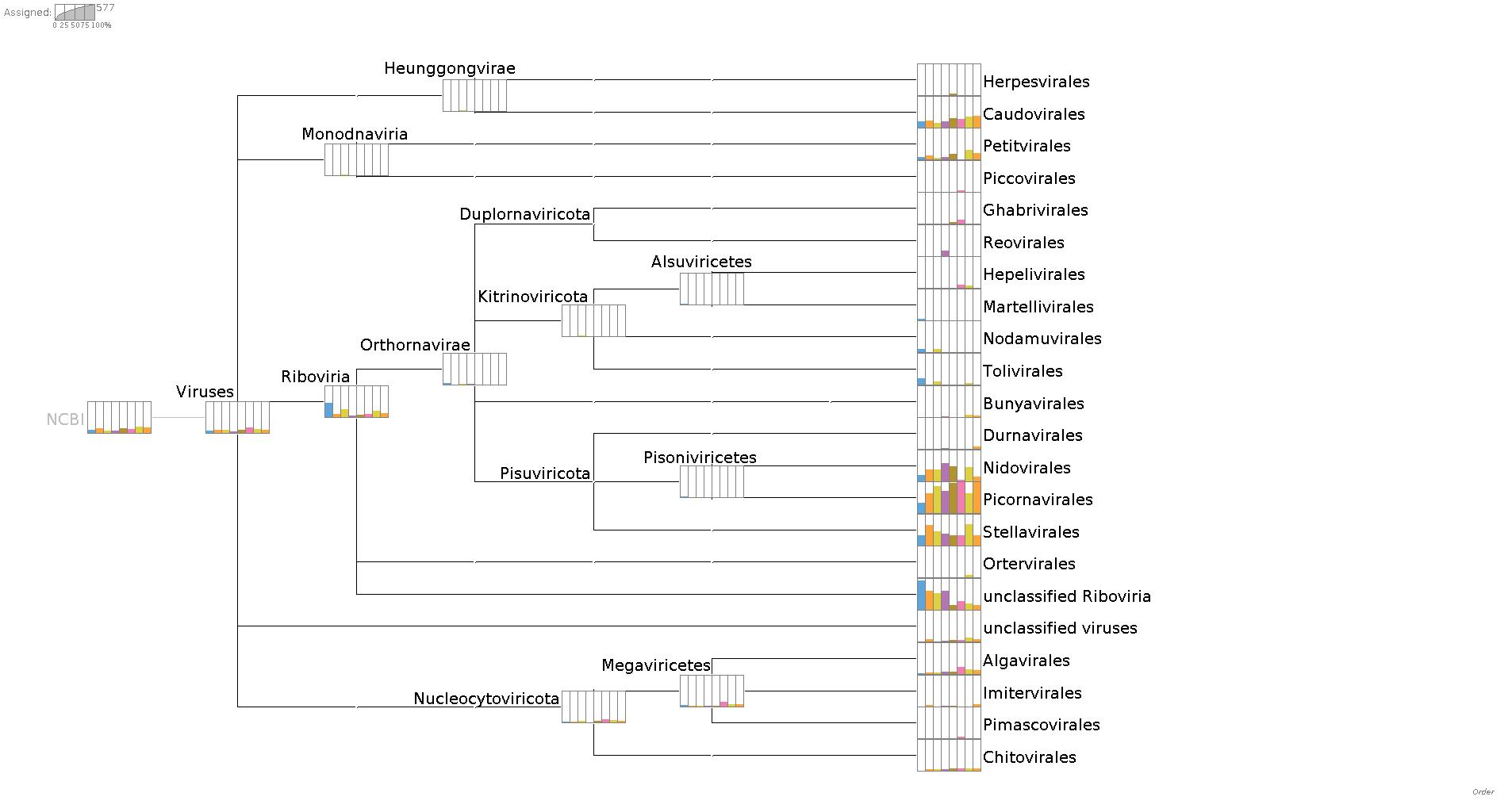


*Figure SF2: Normalized comparison of viral hits obtained after mNGS from F pools F2.1 – F2.8, analyzed with diamond BLASTx algorithm and visualized in MEGAN software. Pools are displayed from F2.1-F2.8 from left to right.*


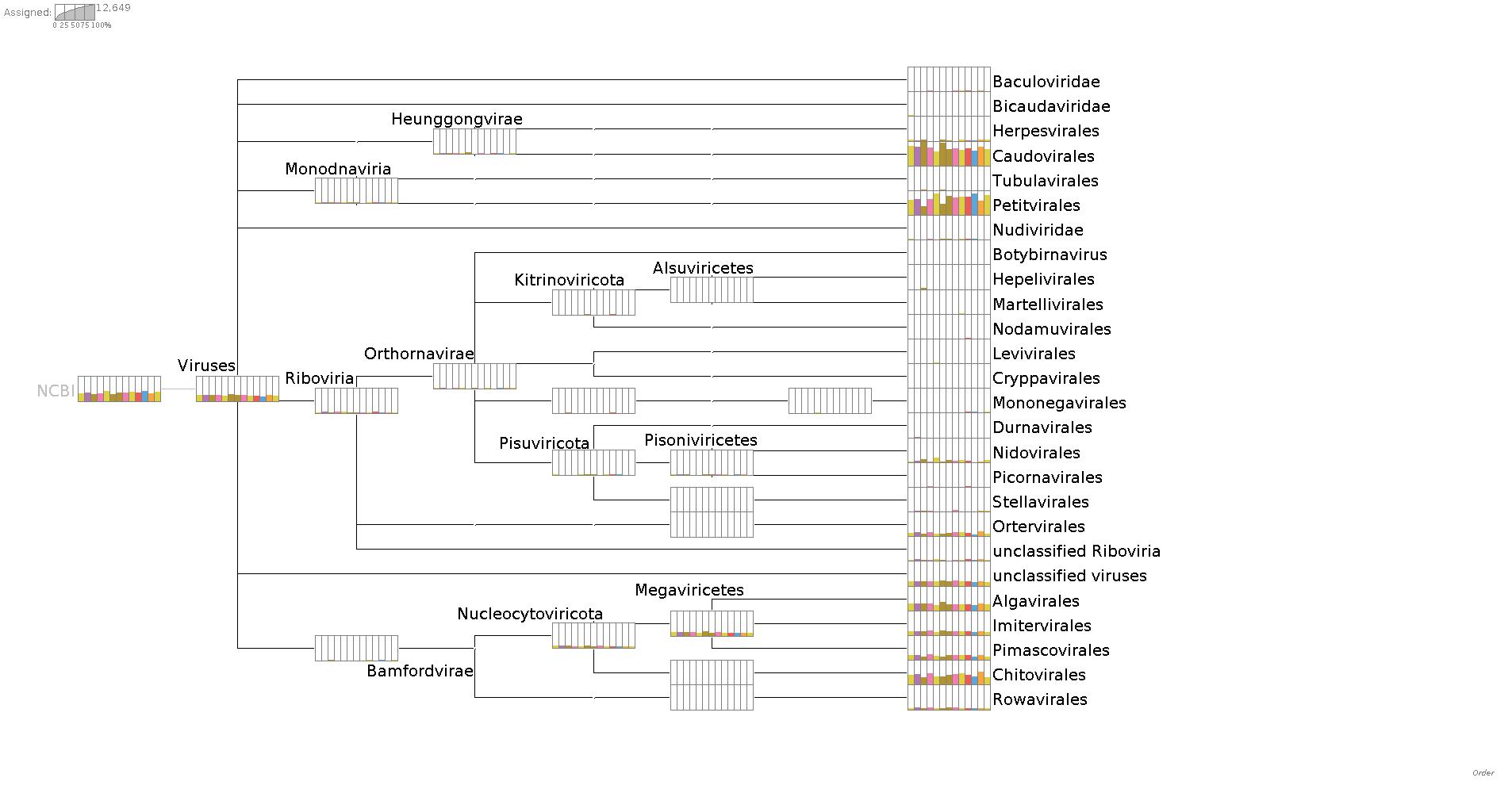


*Figure SF3: Normalized comparison of viral hits obtained after mNGS from US pools US2.1 – US2.14, analyzed with diamond BLASTx algorithm and visualized in MEGAN software. Pools are displayed from US2.1-US2.14 from left to right.*
